# Effects of Expanded Coverage for Chiropractic Services on Medicare Costs in a CMS Demonstration

**DOI:** 10.1371/journal.pone.0147959

**Published:** 2016-02-29

**Authors:** William B. Stason, Grant A Ritter, Timothy Martin, Jeffrey Prottas, Christopher Tompkins, Donald S. Shepard

**Affiliations:** Schneider Institutes for Health Policy, The Heller School, MS 035, Brandeis University, Waltham, Massachusetts, United States of America; University of Michigan, UNITED STATES

## Abstract

**Background:**

Moderately convincing evidence supports the benefits of chiropractic manipulations for low back pain. Its effectiveness in other applications is less well documented, and its cost-effectiveness is not known. These questions led the Centers for Medicaid and Medicare Services (CMS) to conduct a two-year demonstration of expanded Medicare coverage for chiropractic services in the treatment of beneficiaries with neuromusculoskeletal (NMS) conditions affecting the back, limbs, neck, or head.

**Methods:**

The demonstration was conducted in 2005–2007 in selected counties of Illinois, Iowa, and Virginia and the entire states of Maine and New Mexico. Medicare claims were compiled for the preceding year and two demonstration years for the demonstration areas and matched comparison areas. The impact of the demonstration was analyzed through multivariate regression analysis with a difference-in-difference framework.

**Results:**

Expanded coverage increased Medicare expenditures by $50 million or 28.5% in users of chiropractic services and by $114 million or 10.4% in all patients treated for NMS conditions in demonstration areas during the two-year period. Results varied widely among demonstration areas ranging from increased costs per user of $485 in Northern Illinois and Chicago counties to decreases in costs per user of $59 in New Mexico and $178 in Scott County, Iowa.

**Conclusion:**

The demonstration did not assess possible decreases in costs to other insurers, out-of-pocket payments by patients, the need for and costs of pain medications, or longer term clinical benefits such as avoidance of orthopedic surgical procedures beyond the two-year period of the demonstration. It is possible that other payers or beneficiaries saved money during the demonstration while costs to Medicare were increased.

## Introduction

Medicare conducted a payment demonstration for chiropractic services in 2005–2007 that expanded Medicare coverage from “manual manipulation of the spine to correct active subluxations and malfunction” to the full range of diagnostic and treatment procedures for neuromuscular and skeletal (NMS) conditions that chiropractors are trained and legally authorized to perform by the state or jurisdiction in which the treatment is provided. The demonstration responded to requests by the American Chiropractic Association (ACA) and was mandated by Congress [1]. During it, coverage was extended beyond manipulation of the spine to include manipulation of the extremities, physical therapy, interventions such as electrostimulation and ultrasound, evaluation and management (E&M) visits, and diagnostic tests including blood tests, x-rays, computed tomography (CT) scans, and magnetic resonance images (MRIs).

In advocating for the demonstration, the ACA argued that expanded coverage would attract additional patients to chiropractors, reduce out-of-pocket costs for beneficiaries, and have the potential to reduce the total costs of care by reducing the use of pain medications and reducing needs for other medical or surgical treatments for NMS conditions. Concerns over increased costs to Medicare, however, led to requirements that the Secretary of Health and Human Services ensure net budget neutrality by recouping any cost increases from Medicare-certified practicing chiropractors by reducing payments for subsequently billed chiropractic services [1].

This report describes the design of the demonstration and its effects on Medicare payments for beneficiaries who were treated for NMS diagnoses in demonstration or matched comparison areas. Results apply to all Medicare beneficiaries who were treated for NMS diagnoses, though the principal focus is on those who received chiropractic services. Unlike an experiment, the demonstration did not assign experimental and control areas randomly. Instead, the experimental areas were selected by CMS with input from a contractor [2] and the Congress. The comparison areas were selected by the evaluator. Detailed results of the demonstration are presented in the authors’ final report to CMS [3] and summarized in a brief commentary [4].

### Background on Chiropractic

Chiropractic has been characterized as a health care profession “at the crossroads of alternative and mainstream medicine”[5]. Its principal focuses are on the diagnosis, treatment, and prevention of mechanical disorders of the spine and musculoskeletal system under the belief that these disorders affect general health via the nervous system [6]. Core treatments involve manual manipulation of the spine, other joints, and soft tissues with specific focuses on chiropractic subluxations or spinal joint dysfunctions [7, 8], while additional treatments include exercises and health and lifestyle counseling. Though controversial, [9, 10] chiropractic is the largest alternative medical specialty in the United States [11], used by 6% to 12% of U.S. and Canadian populations, including both adults and children [12]. Low back and neck pain are the principal focuses of treatments, though many chiropractors treat a broader spectrum of ailments. Satisfaction with care has been reported to be high and to be strongly related to good communications between chiropractors and their patients [13]. Insurance coverage varies among states and among insurers. Medicare currently limits reimbursement to services that involve manipulation of the spine, while commercial insurers and HMOs often provide broader coverage.

### Evidence on the Clinical Effectiveness of Chiropractic Treatments

Knowledge of the effectiveness of spinal manipulation therapy (SMT) is based on both individual clinical studies and systematic reviews and metanalyses of published studies. Results vary because of variations in study design and small sample sizes [9, 14, 15]. A recent systematic review of reviews concluded that spinal manipulation therapy (SMT) has not been proven to be effective for any condition [16] with the possible exception of low back pain [14]. Support is strongest for the treatment of subacute or chronic low back pain [17] and is less secure for acute low back pain, sciatica, or acute lumbar radiculopathy [18–20]. The effectiveness and cost-effectiveness of maintenance chiropractic care for back pain are not known [21]. Chiropractic treatments of the back appear to be safe [22], though controversy exists over the risk of stroke from cervical manipulation [23]. Limited evidence supports benefits from SMT in the treatment of tension or migraine headaches [24, 25], pain due to knee osteoarthritis [26] treatment of hip osteoarthritis [27], lateral epicondylosis (tennis elbow) [28], and the management of shoulder pain [29].

## Methods

This study was approved by the Brandeis University Committee for the Protection of Human Subjects. The study included all fee-for-service (FFS) Medicare beneficiaries who received services for principal NMS diagnoses in demonstration or matched comparison counties during the two-year period from April 1, 2005 through March 31, 2007. Medicare payments were examined for outpatient services, chiropractic services, and total care (inpatient and outpatient services). In effect, the demonstration focused on ‘intent-to-treat’ populations to examine the effects of increased Medicare coverage for chiropractic services. Data on the use of medications under Part D of Medicare were not available to the analysis. Our evaluation’s final report to Medicare provides further details [3].

### Demonstration Areas

Authorizing Congressional legislation required inclusion of at least four geographic areas–two urban and two rural–with one of each type including a primary care Health Professional Shortage Area (HPSA). Demonstration areas were selected by CMS with inputs from the U.S. Congress and the ACA. States were excluded from participation if their chiropractic practice regulations differed substantially from the norm or if their prior chiropractic utilization, costs, or provider supply were extremely high or low. Two demonstration areas were predominantly urban and two were more rural in character. The selected areas included 26 counties in northern Illinois including the Chicago metropolitan area, Scott County in Iowa, 17 counties in central and northern Virginia, and the full states of Maine and New Mexico.

### Comparison Areas

Comparison areas were counties from adjacent or other nearby states. However, treatment and comparison counties were not permitted to share boundaries in order to minimize boundary crossing by beneficiaries in comparison areas who were seeking the benefits of expanded coverage for services. Comparison areas had to be covered by the same Medicare insurance carrier as the participating area to control for differences in chiropractic claims processing and utilization management procedures. A multi-step process was used to identify comparison counties that matched demonstration counties on key variables associated with chiropractic service utilization and Medicare reimbursement. Correlation analysis was used to identify county-level characteristics that were significantly associated with the volume and costs of Medicare chiropractic service use. These variables included overall Medicare reimbursements per beneficiary, urban/rural status, HPSA/non-HPSA status, and measures of race and socioeconomic status. Principal component analysis was applied to this set of variables and combined with chiropractic reimbursements per beneficiary to construct a one-dimensional factor and summary score for each county. This factor was used to identify closest matches of potential comparison counties to each demonstration county. Each demonstration county was compared with the combined total of two comparison counties to assure sufficiently large sample sizes.

### Study Population

The study included all Medicare beneficiaries in demonstration areas who were 65 years of age or older or were under age 65 and covered by Medicare because of a disability, were residents of a demonstration area, and had at least one paid Medicare claim for a principal NMS diagnosis during the demonstration period from a provider in that area. Comparison groups were like beneficiaries with NMS diagnoses from matched comparison counties.

### Analysis Plan

The analysis relied on Medicare Parts A and B claims for NMS services for Medicare beneficiaries in demonstration and comparison counties for the pre-demonstration year (April 1, 2004 through March 31, 2005) and the two years of the demonstration (April 1, 2005 through March 31, 2007). To ensure that late-submitted claims were included, data were acquired twelve months after the demonstration ended, when datasets are typically at least 99% complete. Descriptive analyses characterize service use and costs, and regression analyses assess the effects of the demonstration while adjusting for differences among regions of the country and patient characteristics. The analysis examines impacts on Medicare payments for all beneficiaries with an NMS diagnosis (All NMS Analysis) and, specifically, for users of chiropractic services (Chiropractic User Analysis). Both approaches account for payments for services related to NMS diagnoses, and further classify services received into chiropractic or other and into “institutional” (Part A, including hospital inpatient, skilled nursing facility and home health agency) or”ambulatory”(mainly Part B and including outpatient, physician, and durable medical equipment). Analyses examine payments by type of NMS diagnosis (spine only, spine and extremities, extremities only, any of these plus a neurological diagnosis), state, urban/rural location, and the HPSA designation of the county in which the treatment was given. All costs and payments are in current (2005–2007) US dollars.

Comparisons between beneficiaries from demonstration and comparison counties are based on a pre-post, treatment-comparison design. Difference-in-difference statistics were constructed from the claims for comparable six-month time periods and were used to estimate the effects of the demonstration on the utilization of services and their costs. Beginning with large cohorts of beneficiaries from demonstration and comparison areas matched on county demographic characteristics and Medicare utilization measures, this approach uses pre-demonstration information to adjust for differences between demonstration and comparison areas’ subjects that may remain despite efforts to match them. Further, it adjusts for changes over time in the use of chiropractic services and other NMS-related treatment issues that are unrelated to the demonstration but may influence its outcomes.

Difference-in-difference statistics are computed on all measures for all beneficiaries with NMS diagnoses—those who received NMS services and those who received chiropractic services under expanded coverage in each of the five market areas. The demonstration’s effects are estimated in terms of the percentage of beneficiaries who used a specific type of service as a proportion of their respective beneficiary samples:

$$demo\;effect\;(proportion)=\left(p_{D,1}-p_{D,0}\right)-\left(p_{C,1}-p_{C,0}\right),$$

where p is the prevalence of service users in the respective areas, subscripts D and C distinguish demonstration and comparison subjects, and subscripts 0 and 1 distinguish between time 0 (pre-demonstration period) and time 1 (demonstration period).

Similarly, analyses of the effects of the demonstration on the number of visits and Medicare reimbursements use ‘difference-in-differences’ effects for continuous measures:

$$demo\;effect\;(continuous)=\left(M_{D,1}-M_{D,0}\right)-\left(M_{C,1}-M_{C,0}\right),$$

where *M* is the mean of the continuous outcome measure of interest (visits or payments), subscripts *D* and *C* distinguish demonstration and comparison subjects, and subscripts 0 and 1 distinguish between time 0 (pre-demonstration period) and time 1 (demonstration period).

To assess budget neutrality, the sign, magnitude, and standard error of the interactions between demonstration status (versus comparison areas) and the indicators for each of the two demonstration years (versus baseline) in the all-NMS user analyses represent the estimated direction, size, and precision of the demonstration’s effect per year for each beneficiary, respectively. Analyses weighted per-person were compared with analyses weighted by county to examine the effects on results of large population centers such as the Chicago, Illinois area.

### Regression Analyses

Hierarchical linear modeling and multivariable regression analysis were used to assess the effects of the demonstration on Medicare payments in demonstration counties in relation to matched comparison counties. The approach is described in detail in the study’s final report to CMS [3]. The model includes individual Medicare beneficiary and county-level covariates and time variables (Year 1 or Year 2 of the demonstration) to adjust for yearly trends with the pre-demonstration year as the reference period). Dependent variables in the analytical model are annual Medicare payments during the pre-demo year (pre) and the two years of the demonstration years (denoted as 1 and 2) for claims with a principal NMS diagnosis for beneficiaries who resided in a demonstration or comparison county. The main outcomes are total Medicare payments for NMS services and Medicare payments for services rendered by chiropractors. To clarify cost differences, subtotals were calculated separately for institutional and ambulatory services. The general form of the model is:

$$y_{i,\;t}=\alpha_{0}+\beta_{1}\;w_{1}+..+\beta_{m}\;w_{m}+\kappa_{1}\;x_{1}+..+\kappa_{n}\;x_{n}+\delta_{1}\;t_{1}+\delta_{2}\;t_{2}+\gamma_{1}\;x_{1}\;t_{1}+\gamma_{2}\;x_{1}\;t_{2}+\varepsilon_{i,\;t}$$

where i denotes the Medicare beneficiary, the w_i_’s are beneficiary characteristics; x_i_’s are characteristics of the county in which the beneficiary resides (including x_1_ which indicates participation in the demonstration); t’s are the time period (year) indicators; and the model contains interactions between demonstration status (x_1_) with time period indicators (t_1_ and t_2_). The ε_i,t_ term represents the individual random error for each beneficiary, i, in each time period t. The hierarchical nature of the model comes from use of the technique of generalized least squares (GLS) to generate unbiased parameter estimates. The letters α, β, κ, δ, and γ denote fixed coefficients estimated by the model. The key coefficients are the interaction terms, γ _1_ and γ _2_, which provide estimates of the differential change in cost per NMS-diagnosed Medicare beneficiary (or chiropractic user) in each year during the demonstration period, after controlling for other factors. The γ_1_ and γ_2_ coefficients represent the estimated “adjusted pre-post difference-in-difference effects of the demonstration” in years 1 and 2, respectively, in dollars per beneficiary per year.

## Results

### Population Characteristics

Demonstration areas included nearly one million Medicare beneficiaries who were being treated for NMS diagnoses, and comparison areas included nearly 600,000 (**Table 1)**. In the year prior to the demonstration, 10.0% of NMS patients in demonstration areas and 13.1% in comparison areas had used chiropractic services. Nearly half of chiropractic service users had also used other types of medical services to treat their principal NMS diagnoses. Among chiropractic users, outpatient visit rates for other types of NMS medical services were somewhat higher in demonstration than matched comparison regions (4.4 vs. 3.7 visits) in the year before the demonstration, while average use of chiropractic services was about equal (10.7 vs. 10.6 visits). During the demonstration period, mean chiropractic visit rates increased more in demonstration than comparison areas (by 1.7 vs. 0.5 visits per year).

**Table 1:** Annual Use Rates of Chiropractic Services by Medicare Beneficiaries with Neuromusculoskeletal (NMS) Diagnoses in Demonstration and Comparison Areas.

			Users of chiropractic services	
Time Period	Number with NMS Diagnoses	Percent Using Chiropractic Services	Visits per Year for Other NMS Services	Visits per Year for Chiropractic Services
Demonstration Areas				
Pre-Year	951,825	10.0%	4.4	10.7
Year 1	994,052	10.8%	4.7	11.5
Year 2	991,265	11.2%	4.8	12.4
Matched Comparison Areas				
Pre-Year	569,704	13.1%	3.7	10.6
Year 1	571,166	12.9%	3.9	10.9
Year 2	566,867	13.0%	4.1	11.1

Source: Authors’ calculations from Medicare claims data.

### Participation of Chiropractors in the Demonstration

Participation of licensed chiropractors ranged from 34% to 56% in different regions and at different times during the demonstration. Higher participation rates were achieved in Maine and Illinois and lower rates were in Central Virginia counties and the state of New Mexico. The most dramatic increases in participation rates during the course of the demonstration were in northern Illinois counties, where rates increased from 39% during the first year to 51% during demonstration’s second year.

### Effects of the Demonstration on Medicare Costs

Medicare payments for the treatment of NMS diagnoses increased by $109 per person and a total of $114 million in all beneficiaries who received treatment for NMS diagnoses in demonstration compared with control areas during the two-year demonstration period (panel for all Medicare beneficiaries in **Table 2**).

**Table 2:** Effects of the Demonstration on Medicare Payments for Beneficiaries with Neuromusculoskeletal (NMS) Diagnoses.

Type of service	Baseline Payments per Person -Year	Increases in Year 1	Increases in Year 2	Total effects per person	Total effects in $ millions
		(SE)	(SE)	(SE)	(SE)
All Medicare Beneficiaries					
Institutional	$470	$32**	$21**	$52**	$55**
		($5)	($5)	($9)	($10)
Ambulatory	$577	$10**	$47**	$56**	$59**
		($3)	($3)	($4)	($5)
All	$1,047	$42**	$67**	$109**	$114
covered services		($7)	($7)	($11)	($12)
Users of Chiropractic Services					
Institutional	$365	$17	$18	$35	$5
		($12)	($12)	($21)	($3)
Ambulatory	$765	$117**	$170**	$287**	$45**
		($7)	($7)	($12)	($2)
All Medicare-	$1,129	$134**	$188**	$322**	$50**
covered services		($16)	($16)	($27)	($4)

Source: Calculations use Medicare claims data. Institutional services are those under Medicare Part A. Ambulatory services are those under Medicare Part B.

Statistical significance is indicated by

* (p<0.05) and

** (p<0.01).

Effects in Year 1 and Year 2 are incremental costs per beneficiary. Components may not add exactly to totals due to rounding.

Notes: Standard errors (SE) are in parentheses

Total increases were $55 million in institutional (Part A) payments and $59 million in ambulatory (Part B) payments. When the analysis is restricted to users of chiropractic services (lower panel of **Table 2**), corresponding increases were $322 per person and $50 million overall. Of the total increase, $45 million was for ambulatory care services of which $35 million was for chiropractic services. Increases in the costs of ambulatory care services in chiropractic service users were 45% greater in Year 2 than Year 1 of the demonstration due to gradual increases in the numbers of participating chiropractors.

### Effects on Chiropractic Costs by Type of Service and Diagnosis

The mean increase of $322 in total Medicare costs per chiropractic user included $224 for chiropractic services and $98 for other medical services. Aggregate cost increases totaled $50 million of which $35 million was for chiropractic services and $15 million for other medical services. The majority of chiropractic service users (61.4%) were treated for conditions that affected both the spine and extremities, while 16.9% of users were treated only for the spine, 20.7% for neuromusculoskeletal diagnoses that included a neurological component such as headache in addition to spine and/or extremity conditions, and 1.1% for diagnoses limited to the extremities. (**Table 3**)

**Table 3:** Increases in Medicare Costs in Chiropractic Users by Type of Service and Diagnosis over the 2 Years of the Demonstration.

Category	% of chiropractic users (N = 155,086)	Cost per user		SE	Aggregate costs (million $)		SE
Type of Service							
Chiropractic services	100.0%	$224		($19)	$35		($3)
Other medical services	100.0%	$98		($8)	$15		($1)
All services	100.0%	$322	**	($27)	$50	**	($4)
Diagnosis							
Spine only	16.9%	$142	**	($16)	$4	**	$0
Extremities only	1.1%	$339		($216)	$0.60		$0
Spine plus extremities	61.4%	$355	**	($35)	$34	**	($3)
Add neurological diagnosis	20.7%	$357	**	($95)	$11	**	($3)
All diagnoses	100.0%	$322	**	($27)	$50	**	($4)

Notes: Standard errors (SE) are in parentheses. Statistical significance is indicated by

* (p<0.05) and

** (p<0.01).

### Results by Market Area

Counties in northern Illinois accounted for two-thirds of all chiropractic users, the largest impact on costs per user ($485), and $49 of the $50 million total increase in Medicare payments (**Table 4)**. Small and statistically insignificant increases in costs per person and total costs occurred in Maine and counties in central and northern Virginia, while small decreases were found in Scott County, Iowa, and New Mexico. The five counties that define metropolitan Chicago accounted for 40% of all chiropractic users, 78% of total cost increases, and an average increase of $632 per user, compared with $232 per user in other northern Illinois counties. The largest increases in cost per chiropractic user were in urban and rural non-HPSA areas ($404 and $249 per user, respectively). Urban non-HPSA areas accounted for over two-thirds of all patients and 84% of the cost increases. Effects on costs in urban and rural HPSA areas were small and not statistically significant.

**Table 4:** Changes in Medicare Costs in Chiropractic Users by Demonstration Area and Type of Market Area.

	% of chiropractic users (N = 155,086)	Cost per user		SE	Aggregate costs (million $)		SE
Demonstration Areas							
Northern Illinois	65.6%	$485	**	($33)	$49	**	($3)
Scott County, IA	4.0%	-$178		($195)	$1		($1)
Maine	12.2%	$35		($105)	$1		($2)
New Mexico	14.0%	-$59		($74)	-$1		($2)
Virginia	4.1%	$136		($106)	$1		($1)
All areas	100.0%	$322	**	($27)	$50	**	($4)
Types of Market Area							
Urban non-HPSA	67.6%	$404	**	($34)	$42	**	($4)
Urban HPSA	0.8%	$97		($195)	$0.10		$0
Rural non-HPSA	26.7%	$249	**	($49)	$10	**	($2)
Rural HPSA	4.9%	$16		($122)	$0.10		($1)
All types	100.0%	$322	**	($27)	$50	**	($4)

Notes: HPSA denotes health provider shortage area. Standard errors (SE) are in parentheses. Components may not add exactly to totals due to rounding. Statistical significance is indicated by

* (p<0.05) and

** (p<0.01).

### Demonstration’s Effects Using County Weighting

**Fig 1:**
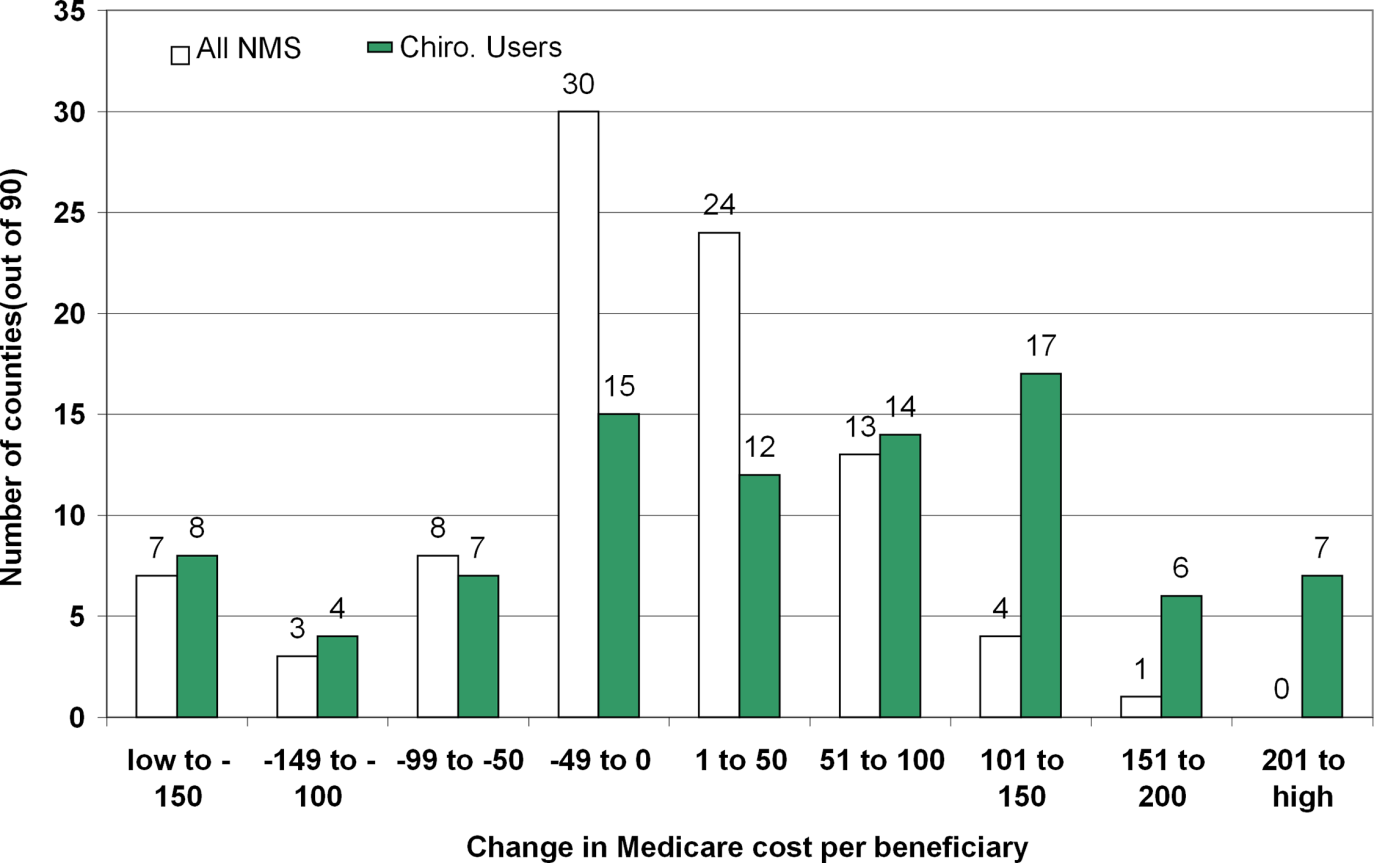
Demonstration Effects on 2-Year Costs per Beneficiary by County.

The analysis of the demonstration’s effects on Medicare costs, to this point, has reflected per-person weighting. This approach attaches more significance to larger population centers and, especially, to the greater Chicago area. County-weighting using equal weights for each county provides an informative alternative. In this analysis, the demonstration’s effects on mean NMS costs and chiropractic costs per person in each demonstration county was compared with its two matched comparison counties. The demonstration included 92 counties in the five demonstration areas. **Fig 1**shows the effects on Medicare costs per beneficiary for 90 of the 92 counties. To make the results more robust, one county at each extreme of the distribution (the upper and lower) were omitted. Costs in chiropractic users showed more consistent increases by county than in all NMS users. Even among chiropractic users, however, decreases in costs were found in 34 of 90 counties during the demonstration.

* The horizontal axis is the net cost per person with a neuromusculoskeletal diagnosis (NMS) diagnosis aggregated over 2 years in 2005–2007 US dollars. Results are categories with a width of $50 per person. The vertical axis is the number of demonstration counties in that category.

Although NMS and chiropractor distributions show twice as many counties with cost increases as decreases, considerable variability was noted among counties. For example, decreases in costs during the demonstration among users of chiropractic were found in over one-third of counties (34 of 90).

In addition to weighting counties equally, two other approaches to county weighting were examined in sensitivity analyses—equal weights per person and equal weights per person with trimming for high population counties. Striking differences in total cost increases from the demonstration were found in the all-NMS user analysis. The smallest increase, $14.7 million, was found when counties were weighted equally; an intermediate result was $65.3 million and the highest value, $79.2 million, arose when counties were weighted by population size. For chiropractic users, however, costs varied only from $12.1 to $15.6 million along the same range of options. Each sensitivity analysis gave lower estimates of cost increases than the values from the main analysis ($114 million in all NMS users and $50 million in chiropractic users).

## Discussion

Medicare’s demonstration of expanded coverage for chiropractic services was authorized by Congress and responded to the ACA’s request for chiropractors to be “treated like other physicians” and to be reimbursed for all services they were trained and legally authorized to provide [1]. It was conducted in four geographic areas and involved nearly one million Medicare beneficiaries with NMS symptoms. Fewer than half of licensed chiropractors in these areas participated in the demonstration. Reasons for low participation included the technical complexity of billing Medicare under it, chiropractors’ concerns about closer monitoring of their practices by Medicare, and the advantages of continuing to bill patients or other payers for their services. Major findings were a $114 million overall increase in Medicare expenditures that included a $50 million increase in the 11% of beneficiaries with NMS problems who received chiropractic care. Interpretation of these findings depends on several factors that characterized the demonstration’s design and assessment of outcomes including the effectiveness of matching between experimental and control counties, possible differences in the baseline severity of symptoms between chiropractic users and non-users, and challenges within the design of the demonstration to attributing clinical improvements to the type(s) of treatments received. Assessment of health care costs was limited to Medicare costs during the two-year demonstration and did not permit assessment of possible cost offsets of the effects of the increased use of chiropractic services on the use and costs of other types of medical services, pain medications, or subsequent musculoskeletal surgical procedures.

Particularly important findings were the large differences in the effects of the demonstration on Medicare costs in different regions of the country and among counties within a given region. For example, counties in northern Illinois accounted for 66% of Medicare participants and 98% of the incremental costs of the demonstration. Increases in the Chicago metropolitan area were especially dramatic. By comparison, chiropractic costs increased only modestly in Maine and in counties of north-central Virginia and actually decreased in Scott County, Iowa and the state of New Mexico. When the analysis was weighted by county, chiropractic costs decreased in 35 of 98 (36%) of participating counties. Site visits by the investigators to northern Illinois were impressive for the high degree of interest of chiropractors in the demonstration, how well Chicago area chiropractors had prepared for it, and concerns of chiropractors over the delays in implementation that occurred during its first year [3]. These findings were supported by separate analyses that found widespread geographic variations in chiropractor’s practice behaviors and associated costs [30, 31]. Sensitivity analyses suggest that cost increases would have been less dramatic if the demonstration sites had been more representative of practice patterns in the country as a whole and of more equal population sizes. These changes would have reduced the likelihood that one site would dominate the results so significantly.

The all-NMS user and chiropractic user analyses provide complementary perspectives. The former was well-suited to identifying unintended effects of the demonstration such as possible increases in the use and costs of health services due to redundancy of care between chiropractors and other physicians. Analyses focused on chiropractic users, on the other hand, provide direct evidence of changes in patterns and costs of services that resulted from expanded Medicare coverage of chiropractic services.

Expanded coverage for chiropractic services clearly increased Medicare payments to chiropractors. Less certain, however, is how much of the increase was due to real increases in the use of chiropractic services versus cost-shifting to Medicare for services that were previously paid “out-of-pocket” by beneficiaries or were being reimbursed by other insurers. More than two-thirds of demonstration participants reported having supplemental health insurance that included at least some coverage for chiropractic services [3]. Hence, cost-shifting to Medicare may well have played an important role. The 10% net increase in patients with NMS diagnoses who used chiropractic services during the demonstration may reflect increased patient demand for chiropractic services or increased billing of Medicare by chiropractors who had previously billed other insurers or patients who were paying “out of pocket” for their services. For the demonstration to have achieved budget neutrality for Medicare, increased billing for chiropractic services would need to have been offset by near-term reductions in the use of inpatient or non-chiropractic outpatient services. No such offsets were observed. Potential effects in reducing the use of pain or other medications could not be examined because Medicare data on medication costs (Part D) were not available to the investigators during the evaluation.

### Strengths of the Demonstration

The demonstration was conducted in multiple, geographically dispersed states and included more than a million Medicare beneficiaries with NMS diagnoses who were potential candidates for chiropractic treatments. These factors lend some support to the national representativeness of the sample. Other strengths were that the demonstration design and evaluation went to considerable efforts to match experimental and comparison counties on Medicare Administrative Contractor (carrier) and other attributes that were likely to influence the use of chiropractic and medical services. Use of hierarchal modeling and regression analyses made the best possible use of these matching variables in assessing the impacts of the demonstration and adjusting for possible group differences. Inclusion of baseline county characteristics in the regression model helped to adjust for differences between the demonstration and comparison counties.

### Weaknesses of the Demonstration

One important concern is whether participating demonstration areas were representative of chiropractic practices throughout the U.S. This concern is underscored by the small number of market areas included and the fact that two-thirds of chiropractic users lived in a single market area–selected counties in northern Illinois that included the metropolitan area of Chicago. A second concern is the fact that fewer than half of eligible chiropractors in the demonstration areas participated in the demonstration. Medicare’s complex billing requirements and chiropractor’s concerns about its close surveillance of chiropractic practices were among the reasons given by chiropractors for low participation rates. Other factors included easier billing and higher reimbursement rates by private insurers or self-pay patients. For this range of reasons, participating chiropractors may not have been representative of all chiropractors.

The brief two-year duration of the demonstration was an important weakness in its design that left open critical questions about whether longer-term increases in access to chiropractic treatments would help to reduce health care costs by reducing levels of disability and needs for neuromuscular disease related surgery. Other weaknesses were that cost offsets from savings to other insurers, reduced use of pain medications, reduced out-of-pocket patient-borne costs were not available to the analysis. Cost offsets from these sources may well be important.

### Conclusions

Expanded coverage of chiropractic services in the demonstration increased Medicare expenditures by $50 million or 28.5% in users of chiropractic services and by $114 million or 10.4% in all patients treated for NMS conditions in demonstration areas during the two-year period. Effects varied widely among demonstration areas and ranged from increased costs per user of $485 in Northern Illinois counties including Chicago to decreases in costs per user of $59 in New Mexico and $178 in Scott County, Iowa. These variations parallel substantial geographic variations found by other researchers [32].

Limitations of the demonstration design are important. These include questions about the representativeness of experimental sites, its relatively short two-year duration, and design features that prevented assessment of effects on costs to insurers other than Medicare, out-of-pocket payments by patients, effects on the use and costs of pain medications, or longer term clinical benefits such as possible reductions in needs for orthopedic surgical procedures beyond the period of the demonstration. More research, including controlled multi-site, multi-year, and multi-payer studies will be needed to obtain robust evidence on the clinical benefits, costs, and possible cost offsets of chiropractic services [33]. A more limited expansion of chiropractor services with limits on utilization might achieve the desired benefits without large cost increases [30]. This demonstration provided useful insights but left open important questions about the ultimate effects of expanded Medicare coverage for chiropractic services in the United States.
